# Assessment of Water Quality among Handwashing and Drinking Water Stations in Schools in Belize, 2022

**DOI:** 10.4269/ajtmh.24-0125

**Published:** 2025-04-08

**Authors:** Anh N. Ly, Alexandra Kossik, Ary Sosa, Uriel Sosa, Dian Maheia, Yolanda Gongora, Russell Manzanero, Francis Morey, Melissa Diaz-Musa, Dennis Nichols, Adrianna Maliga, Kelsey McDavid, Christina Craig, Gerhaldine Morazan, Matthew Lozier, Kristy O. Murray

**Affiliations:** ^1^ Baylor College of Medicine and Texas Children’s Hospital, Houston, Texas;; ^2^ Division of Foodborne, Waterborne, and Environmental Diseases, Centers for Disease Control and Prevention, Atlanta, Georgia;; ^3^ Belize Ministry of Health and Wellness, Belize City, Belize;; ^4^ Culture, Science and Technology, Belize Ministry of Education, Belize City, Belize

## Abstract

Water quality assessments are critical for ensuring timely responses to water-related concerns, particularly in low-resource areas with limited water, sanitation, and hygiene (WASH) infrastructure. In collaboration with the Belize Ministry of Health and Wellness and the Ministry of Education, Culture, Science and Technology, we conducted a survey on WASH infrastructure and resources among 221 schools. We identified 65 schools across all six districts of Belize for water quality testing. Among these 65 schools, 83% had at least one water sample that did not meet the WHO’s recommended free chlorine residual level for drinking water. Additionally, coliforms and *Escherichia coli* were detected in at least one drinking or handwashing water sample from 43 (66%) and 14 (22%) schools, respectively. These findings underscore the importance of routine water quality testing in schools to inform timely public health responses.

Diarrheal disease is a leading cause of morbidity and mortality among children. Globally, there are ∼1.7 billion cases of childhood diarrhea and 525,000 deaths annually.^1^ An estimate from 2016 showed that ∼36% of the diarrheal burden is attributable to inadequate water quality in low- and middle-income countries.^2^ Access to clean water is essential for preventing the spread of gastrointestinal infections.

The United Nations Sustainable Development Goal 6 aims to provide clean water and sanitation for all; however, current efforts related to drinking water need to increase six-fold to achieve this goal by 2030.^3^ As of 2022, 2.2 million individuals globally lacked safe drinking water,^3^ and in 2019, 31% of schools worldwide did not have access to basic drinking water services.^4^

Our study focused on water quality in schools in Belize, a country in Central America bordering Mexico, Guatemala, and the Caribbean Sea. Although the World Bank has recently classified it as upper-middle-income,^5^ there is a stark income disparity in the country, with 35.7% of the overall population living in poverty; this figure can reach as high as 60.3% among populations in the southernmost district of Toledo.^6^ These impoverished communities are at heightened risk of infectious diseases due to a lack of access to resources, including water, sanitation, and hygiene (WASH) services.

A 2009 survey in Belize showed that one in four schools reported having untreated water.^7^ There are currently no national water quality assessments in Belizean schools. In collaboration with the Belize Ministry of Health and Wellness (MoHW) and the Ministry of Education, Culture, Science and Technology (MoECST), we conducted water quality testing across schools in all six districts of Belize.

From December 2021 to January 2022, an electronic baseline survey was distributed to government and government-aided primary and secondary schools in Belize. The survey captured school characteristics, water sources, and hand hygiene infrastructure and resources. Based on the survey results, 54 schools were selected proportionally according to a sampling weight derived from the reported water source (piped versus not piped), perceived water quality concerns (from the question “Are there ever concerns about the quality or cleanliness of water at the school?”), and the rural or urban classification of the schools. Higher priority for selection was given to schools with non-piped water sources, reported water quality concerns, and rural locations. We included 12 additional schools in the water quality assessment as they were selected in another arm of the study based on high needs for hand hygiene resources, and another 12 schools as alternatives in case the schools on the primary list could not be reached for data collection.

In March 2022, study team members visited schools to collect water samples from handwashing and drinking water stations, including piped water, hand pumps, and rainwater collection tanks. Both schools on the original list and some schools on the alternative list were visited. Water samples were initially tested for free chlorine residual using the Hach® Chlorine Color Disc Test Kit (Hach® 223102, Loveland, CO). If the free chlorine residual level was below 0.2 mg/L, the WHO-recommended free chlorine residual level for drinking water,^8^ a 100 mL sample was collected and sent to the National Drinking Water Quality Laboratory (NDWQL) in Belize City for additional testing for coliforms and *Escherichia coli* (*E. coli*). A maximum of five water samples were collected from different access points at each school to accommodate the laboratory’s daily processing capacity. A total of 167 water samples were tested for coliforms and *E. coli*.

Water samples were collected, stored at 4°C, and delivered to the laboratory within 24 hours for processing. Samples were processed using the U.S. Environmental Protection Agency-approved Hach® Company Method 10029, which consists of membrane filtration through a 0.45 µm filter, followed by a 24-hour incubation on m-ColiBlue24 media (Hach® 2608450). Colonies were then enumerated, with red and blue colonies interpreted as total coliforms and blue colonies alone interpreted as *E. coli*.

Descriptive statistics were calculated for school characteristics, perceived water quality concerns, and the main water source used at each school, as reported in the baseline survey. *E. coli* results were aggregated and mapped by district. Statistical analyses were performed using STATA version 16 (StataCorp, College Station, TX), and maps were created using ArcGIS Pro version 3.1.0 (Esri, Redlands, CA).

Among the 308 government and government-aided primary and secondary schools, 221 (72%) responded. The target sample size was 66 schools, and 65 schools were visited for water quality assessment. These 65 schools were distributed across all six districts of Belize and varied in size, with 69% classified as rural (Table 1). In the baseline survey, 29% of all schools expressed concerns about the quality or cleanliness of the water at the school, whereas 45% of the schools selected for assessment expressed similar concerns. As intended, schools that expressed concerns about the quality of water were more likely to be selected (odds ratio = 2.7; 95% CI = 1.45–4.97).

**Table 1:** Characteristics of schools included in the water quality assessment and all schools that responded to the baseline survey

Variable	All Schools in Baseline Survey (*N* = 221), *n* (%)	Schools Selected for Water Quality Assessment (*n* = 65), *n* (%)	Schools not Included in Water Quality Assessment (*n* = 156), *n* (%)	Odds of Selection for Assessment (95% CI)	*P*-Value
School classification					
Rural	146 (66)	45 (69)	101 (65)	Ref	–
Urban	75 (34)	20 (31)	55 (35)	0.82 (0.44–1.52)	0.521
Region (district)					
Northern (Corozal, Orange Walk)	79 (36)	20 (31)	59 (38)	Ref	–
Central (Belize and Cayo)	82 (37)	22 (34)	60 (38)	1.08 (0.53–2.19)	0.827
Southern (Stann Creek, Toledo)	60 (27)	23 (35)	37 (24)	1.83 (0.89–3.79)	0.102
School size					
Small (<100 students)	52 (24)	19 (29)	33 (21)	Ref	–
Medium (100–299 students)	100 (45)	24 (37)	76 (49)	0.55 (0.26–1.14)	0.106
Large (≥300 students)	69 (31)	22 (34)	47 (30)	0.81 (0.38–1.74)	0.593
Perceived water quality concerns*	65 (29)	29 (45)	36 (23)	2.69 (1.45–4.97)	0.002
Main reported source of water at school					
Non-piped	28 (13)	10 (15)	18 (12)	Ref	Ref
Piped	193 (87)	55 (85)	138 (88)	0.72 (0.31–1.65)	0.435

Ref = referent group.

* Schools were classified as having perceived water quality concern if they answered “Yes” to the question “Are there ever concerns about the quality or cleanliness of water at the school?”. This response was collected from the baseline survey before any water quality assessments were conducted by the study team during the data collection period.

Among the 65 schools included in the water quality assessment, most (*n* = 54; 83%) had at least one water sample with a free chlorine residual level below 0.2 mg/L, 43 (66%) had a sample positive for total coliforms, and 14 (22%) had *E. coli* detected in at least one water sample (Table 2). Almost all schools where *E. coli* was detected (13/14; 93%) were classified as rural. The proportion of schools in each district with at least one water sample positive for *E. coli* is shown in Figure 1. The southernmost district of Toledo had the highest proportion of schools with *E. coli* detected in the water, followed by Cayo and then Orange Walk.

**Table 2:** Comparison of water quality assessment results and school characteristics

	Schools with ≥1 Water Sample with Free Chlorine Residual <0.2 mg/L, *n* (%)	Schools with ≥1 Water Sample with Total Coliforms, *n* (%)	Schools with ≥1 Water Sample with *Escherichia coli*, *n* (%)
Total schools	54 (83)*	43 (66)*	14 (22)*
School classification			
Rural	41 (76)	34 (79)	13 (93)
Urban	13 (24)	9 (21)	1 (7)
District			
Belize	8 (15)	6 (14)	1 (7)
Cayo	8 (15)	6 (14)	4 (29)
Corozal	6 (11)	5 (12)	0 (0)
Orange Walk	11 (20)	9 (21)	2 (14)
Stann Creek	10 (19)	6 (14)	1 (7)
Toledo	11 (20)	11 (26)	6 (43)
School size			
Small (<100 students)	19 (35)	17 (40)	5 (36)
Medium (100–299 students)	17 (31)	14 (33)	4 (29)
Large (≥300 students)	18 (33)	12 (28)	5 (36)
Perceived water quality concerns^†^	28 (52)	24 (56)	13 (93)

* Percentage of total schools included in water quality testing (*N* = 65).

^†^ Schools were classified as having perceived water quality concern if they answered “Yes” to the question “Are there ever concerns about the quality or cleanliness of water at the school?”. This response was collected from the baseline survey before any water quality assessments were conducted during the data collection period.

**Figure 1.**
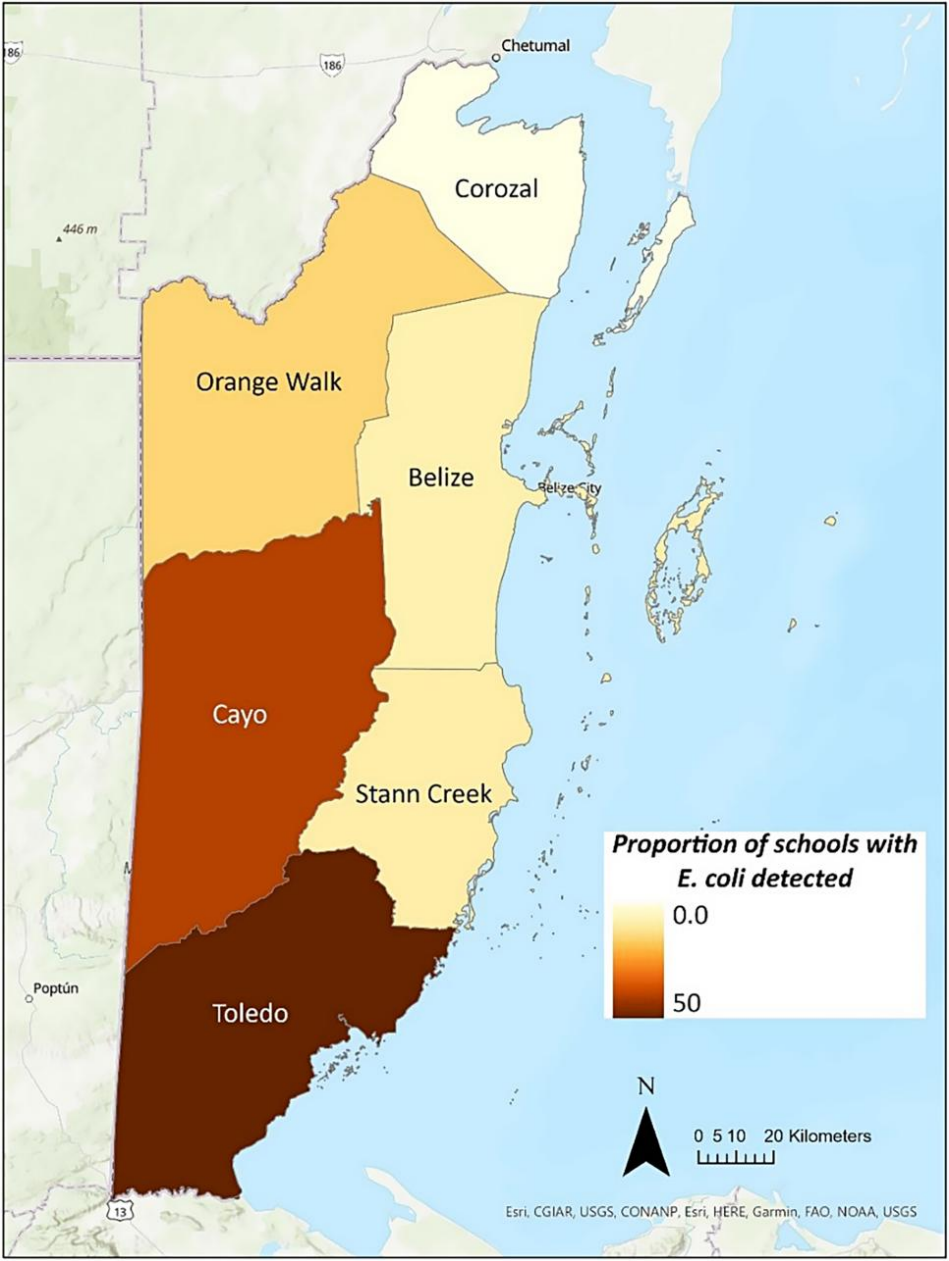
Proportion of schools in each district with *Escherichia coli* detected in at least one handwashing or drinking water sample.

The results of free chlorine residual, total coliforms, and *E. coli* were shared with the MoECST and the Epidemiology Unit of the MoHW. The NDWQL notified personnel within the Ministry of Rural Transformation, Community Development, Labor, and Local Government about the *E. coli*-positive results. The local water boards were alerted and provided with guidance for interventions. The water boards notified the community, including schools, about the water testing results.

For schools with positive *E. coli* results, in accordance with the protocol from the MoHW, water systems were flushed with superchlorinated water to achieve a concentration greater than 5 mg/L. These water systems were inspected for necessary maintenance. Water samples from these schools were then retested by the NDWQL, and additional cleaning of the systems was performed if the results remained positive. The MoHW also developed a plan to perform routine testing at the 14 schools where *E. coli* was detected.

The high rate of contamination in Toledo can be attributed to the economic and geographic characteristics of the district. Toledo has a higher poverty rate than other districts in Belize.^6^ Additionally, many schools in Toledo are located in geographically remote areas, which hinders the frequent collection of water samples for laboratory testing and quality assurance.

Previous studies have found high levels of *E. coli* in drinking water in the Stann Creek and Toledo Districts. A study conducted in three Mayan villages in the Toledo District showed that 46–62% of the collection sites had *E. coli* contamination in drinking water sources.^9^ A field test in the Stann Creek District found that *E. coli* was present in drinking water at the sources (36%) and in households (47%).^10^ To our knowledge, our study is the first to conduct a water quality assessment in schools across all districts of Belize.

One major strength of our study is the inclusion of many schools in remote areas that are difficult to reach for routine assessments. Furthermore, all laboratory analyses and interventions were led by Belizean governmental entities, which is critical for the long-term sustainability of public health interventions. In Belize, community water sources are part of the routine quality monitoring program conducted by the NDWQL. However, during the coronavirus disease 2019 pandemic, monitoring activities were minimized due to staffing and resource constraints. This project helped to expand the water quality monitoring capacity of the MoHW by providing laboratory supplies and assistance with the field collection of water samples.

There are also limitations worth noting. Water samples were collected from multiple source points at each school; unfortunately, the specific water tap was not documented. As a result, the contaminated specimen could not be traced back to its original source. Additionally, only a limited number of schools were included in the study to accommodate laboratory capacity and the restricted time allotted for sample collection at the schools. Because the national survey was conducted electronically, the data may not be representative of the entire country, and the sample of schools selected for water quality assessments may not be representative of each district. Lastly, there are additional water quality components that we did not assess, such as chemicals, heavy metals, and other waterborne microorganisms.

This study highlights the importance of collaboration among public health partners to advance WASH efforts in community settings. The initiatives undertaken by the Belizean government served as an exemplary public health response to address local needs quickly. Although immediate actions were implemented, permanent sustainable solutions are required to ensure that water boards are equipped with the necessary WASH infrastructure and capacity for continuous water quality assessment and monitoring.
